# *Helicobacter pylori* patient isolates from South Africa and Nigeria differ in virulence factor pathogenicity profile and associated gastric disease outcome

**DOI:** 10.1038/s41598-020-66128-0

**Published:** 2020-07-10

**Authors:** Pia Palamides, Tolulope Jolaiya, Ayodeji Idowu, Eva Loell, Charles Onyekwere, Rose Ugiagbe, Ifeanyi Agbo, Olufunmilayo Lesi, Dennis Ndububa, Olusegun Adekanle, Manuel Carranza, Reidwaan Ally, Henry Njom, Isaac A. Adeleye, Ute Harrison, Anna Clarke, Wolfgang Fischer, Stella Smith, Rainer Haas

**Affiliations:** 1 Chair of Medical Microbiology and Hospital Epidemiology, Max von Pettenkofer-Institute, Faculty of Medicine, LMU Munich, Munich, Germany; 2 Department of Microbiology, University of Lagos, Akoka, Yaba Lagos, Nigeria; 3 Department of Biochemistry and Microbiology, Faculty of Science and Agriculture, University of Fort Hare, Alice, 5700 Eastern Cape South Africa; 4 Department of Medicine, Lagos State University Teaching Hospital, Ikeja, Nigeria; 5 Department of Medicine, University of Benin Teaching Hospital, Benin, Nigeria; 6 Department of Medicine, Lagos University Teaching Hospital, Idi-Araba, Nigeria; 7 Department of Medicine, Obafemi Awolowo University Teaching Hospital Complex, Ile-Ife, Nigeria; 8 Institute for Pathology, Faculty of Medicine, LMU Munich, Munich, Germany; 9 Division of Gastroenterology, Chris Hani Baragwanath Academic Hospital (CHBAH), Soweto, Johannesburg 2013 South Africa; 10 Molecular Biology and Biotechnology Department, Nigerian Institute of Medical Research, Yaba, Lagos Nigeria; 11 German Center for Infection Research (DZIF), LMU Munich, Munich, Germany

**Keywords:** Biochemistry, Cell biology, Microbiology, Cancer, Gastrointestinal diseases, Infectious diseases

## Abstract

*Helicobacter pylori* is a gram-negative, spiral-shaped bacterial pathogen and the causative agent for gastritis, peptic ulcer disease and classified as a WHO class I carcinogen. While the prevalence of *H. pylori* infections in Africa is among the highest in the world, the incidence of gastric cancer is comparably low. Little is known about other symptoms related to the *H. pylori* infection in Africa and the association with certain phenotypes of bacterial virulence. We established a network of study sites in Nigeria (NG) and South Africa (ZA) to gain an overview on the epidemiological situation. In total 220 isolates from 114 patients were analyzed and 118 different patient isolates examined for the presence of the virulence factors *cagA, vacA, dupA*, their phylogenetic origin and their resistance against the commonly used antibiotics amoxicillin, clarithromycin, metronidazole and tetracycline. We report that *H. pylori* isolates from Nigeria and South Africa differ significantly in their phylogenetic profiles and in their expression of virulence factors. VacA mosaicism is intensive, resulting in m1-m2 *vacA* chimeras and frequent s1m1 and s1m2 *vacA* subtypes in hpAfrica2 strains. Gastric lesions were diagnosed more frequent in Nigerian versus South African patients and *H. pylori* isolates that are resistant against one or multiple antibiotics occur frequently in both countries.

## Introduction

*H. pylori* is a gram-negative, spiral-shaped, highly motile bacterium that colonizes the stomach of 50–80% of people around the globe^1,2^. It is acquired during early childhood and usually persists for lifetime^3^. Prevalence varies between industrial and developing countries and is influenced by multiple factors as age, host genetic predisposition, sanitation, dietary and socioeconomic factors^1,4^. While the infection remains asymptomatic in most infected people, it can progress to gastritis, peptic ulcer disease and furthermore is a risk factor for the development of gastric cancer and MALT lymphoma^5,6^.

It has been reported that a substantial discrepancy exists between the *H. pylori* prevalence and gastric cancer incidence in sub-Saharan Africa, termed the “African enigma”^7^. Similar phenomena have also been described for other countries with a often lower state of development as India, Bangladesh and Pakistan or even Brasil^8,9^. While the prevalence for *H. pylori* infection in such countries is exceptionally high (estimated prevalence for Nigeria 87.7% and for South Africa 77.6%^2^, the rate of complications is usually comparably low.

In addition to host susceptibility or environmental and dietary impacts, multiple bacterial virulence factors have been studied in the context of disease progression. The most important is the cytotoxin-associated-gene pathogenicity island (*cag*PAI) that harbors a type IV secretion system (T4SS) that injects the effector protein CagA into gastric epithelial cells, where it is phosphorylated by Src kinases at cognate EPIYA motifs^10,11^. Certain forms of EPIYA motifs and the presence of *cagA* itself, have been reported to be associated with a higher pathology^12–14^.

Apart from CagA, also the vacuolating cytotoxin A (VacA) exhibits cytotoxic and immunomodulatory functions^15,16^. It is a pore-forming toxin, which induces the formation of vacuoles in gastric epithelial cells and can cause apoptosis^17,18^. The toxin is about 88 kDa in size and is structured into a signal (s), intermediate (i) and middle (m) region^19^. Different *vacA* variants have been statistically associated with pathologic features; for example, the variant s1m1 has been linked to a more severe pathology^14,20^. While almost all isolates from West Africa or East Asia express the s1m1 variant, the more archetypical type-2 isolates are known to lack the *cagA* gene and express an s2m2 *vacA* gene^21,22^.

A virulence factor that was specially linked to gastric ulceration is the duodencal ulcer promoting gene (*dupA*). Since the gene was first described in 2005 multiple studies with contradictory results about the role of *dupA* in pathogenesis have been published^23–27^. The *dupA* gene is part of a type IV secretion system located on an integrating conjugative element (ICEHptfs4). It has been previously shown that isolates of West African origin have a specific composition with an ICEHptfs4a-variant at the left junction and an ICEHptfs4b-variant homolog at the right junction (ICEHptfs4a/b, also recently designated as L2C1R1^28^). A high proportion of West African isolates also showed a truncation close to the right junction(L2C1R1f)^28^, that was not found in isolates from European origin^29^.

Antibiotic resistance is a rising problem, causing eradication therapy failure for *H. pylori* worldwide. The problem is especially high in countries with limited access to a professional health care system and prescription-free sale of antimicrobial substances. In Nigeria and South Africa two therapy regimens are mainly used: a triple therapy, consisting of a proton pump inhibitor and two antibiotics (amoxicillin and clarithromycin, or metronidazole and clarithromycin) or a quadruple therapy that includes a proton pump inhibitor, bismuth and two antibiotics (amoxicillin and clarithromycin, or metronidazole and tetracycline)^6^. There is an urgent need to adjust standard therapy protocols according to the resistance situation. We therefore tested *H. pylori* isolates for resistance against the four commonly used antibiotics: amoxicillin, clarithromycin, metronidazole and tetracycline.

This work aims to gain more insight into *H. pylori* infections in Nigeria and South Africa, the virulence pattern of the respective isolates and their resistance to commonly used antibiotics. Therefore, we established a network of study sites and cooperation partners in Nigeria and South Africa. Isolates, patient questionnaires, gastroenterological and histopathological diagnosis from 1121 *H. pylori* participating patients have been evaluated, resulting in an extended overview about *H. pylori* infections in West- and South Africa.

## Results

### Study design and demographic data

**Figure 1:**
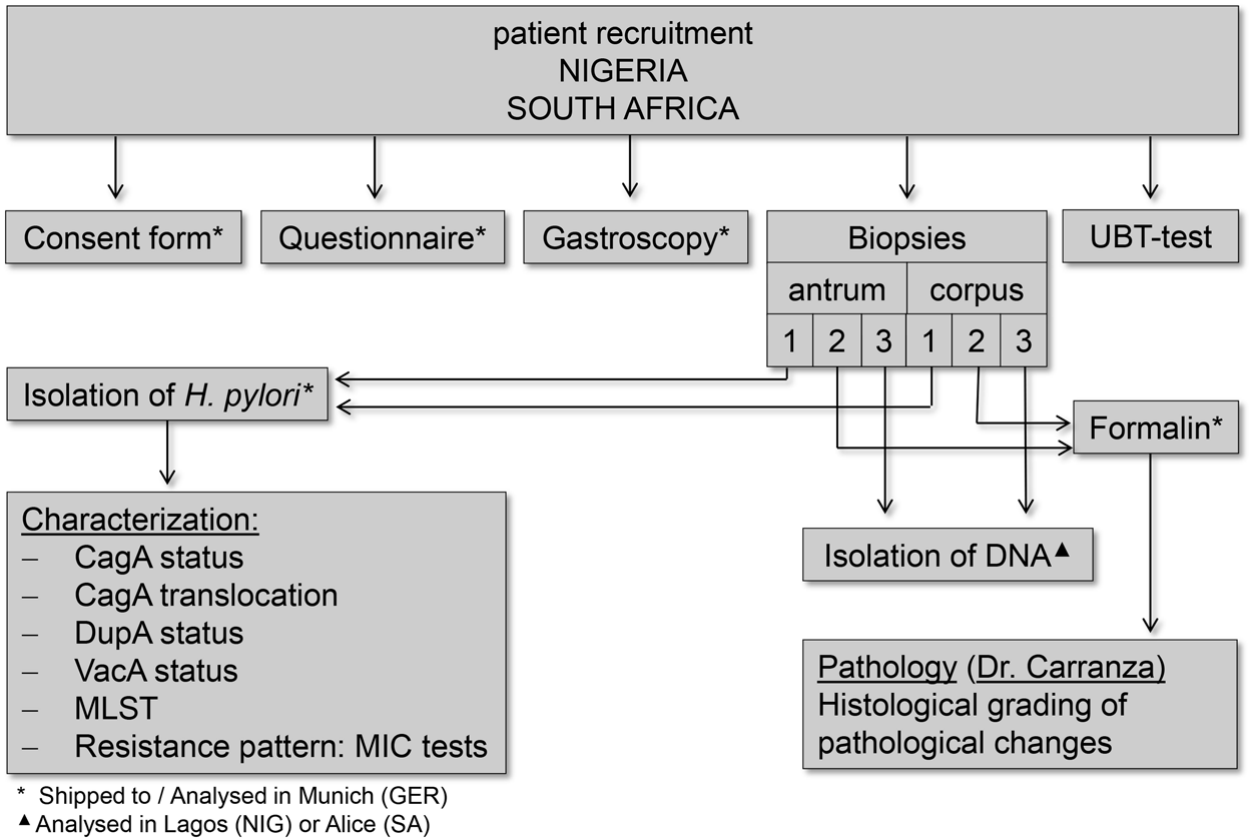
Schematic overview of the study design. Patients were recruited in the Gastroenterology departments of eight different hospitals in Nigeria and South Africa. During gastroscopy diagnosis was noted and biopsies taken. From the biopsies *H. pylori* was cultured, DNA isolated and one biopsy of antrum and corpus fixed for pathohistological analysis.

**Figure 2:**
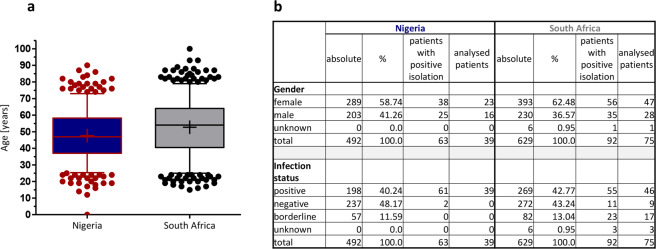
Demographic data of participating patients. (**a**) Depicted are box and whisker plots (5–95% percentile) for the age distribution of the study population at the time of gastroscopy. (**b**) The table shows the demographic distribution of gender and infection status within the study population. Infection status was determined via UBT in Nigeria and with 16 s rRNA PCR in South Africa.

Throughout the course of this study, 492/629 (NG/ZA) patient questionnaires, 486/622 forms with gastroenterological results and 88/132 isolates (from 39/75 patients) were obtained for further analysis, the workflow of which is summarized in Fig. 1. The age distribution of the Nigerian and South African cohorts and demographic data of all patients contributing to the study are summarized in Fig. 2a,b. Patients with either a positive result in UBT (test) or a positive PCR result in antrum and corpus biopsy were considered to be *H. pylori* positive. Whereas the UBT test was considered as the first-line *H. pylori* diagnostics tool, the use of a different method was necessary in South Africa (PCR detection of *H pylori* DNA) due to the restricted access of the UBT kits during the sampling phase. We compared, however, the accuracy and reliability of the two methods for 113 patients, for which both test results (UBT and PCR) were available. Compared to the UBT test, no positive patients remained undetected by PCR, but 2.65% of patients with negative UBT tests were detected to be *H. pylori* positive (positive PCR result in antrum and corpus). From these 113 patients 10 patients (8.85%) with negative UBT result showed a positive PCR result in either antrum or corpus and were therefore classified as borderline positive (Fig. 2b). We therefore considered the PCR detection of *H. pylori* for South African patients as coequal to the UBT method.

We found 2/11 (NG/ZA) patients with negative diagnosis and 0/23 (NG/ZA) patients with a borderline disease status, but successful *H. pylori* reisolation. For statistics that included the full dataset of all patients, these patients were considered as infected, while all other patients with borderline disease status were excluded from the analysis. The mean age at the time of gastroscopy was 47.79 ± 14.71 in Nigeria and 52.69 ± 16.37 in South Africa (Fig. 2b).

### Endoscopic observations and histopathological results show different patterns of disease in Nigeria and South Africa

**Figure 3:**
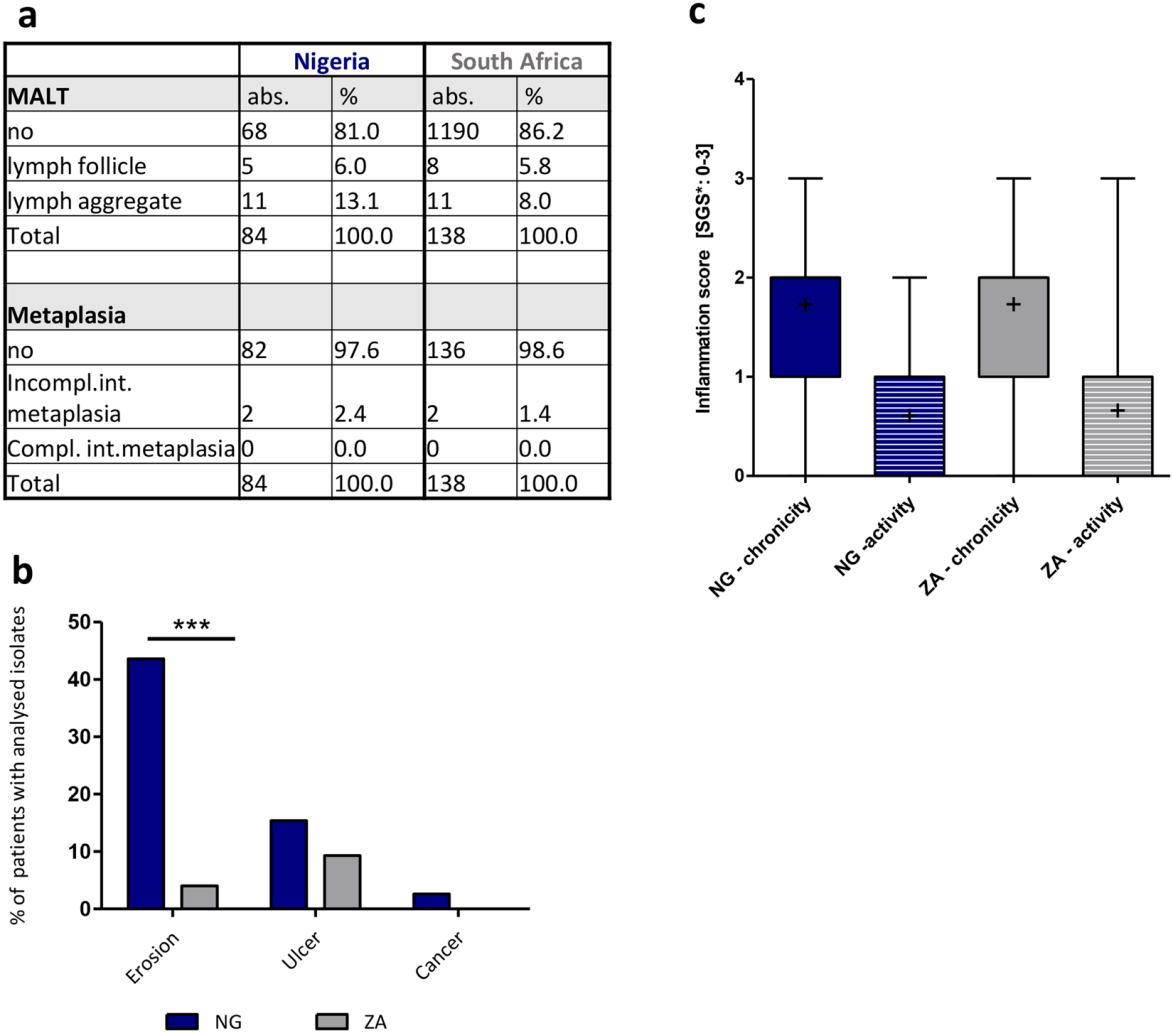
Comparison of gastroenterological and histopathological diagnosis of patients from Nigeria and South Africa. (**a**) The table shows the occurrence of different forms of MALT (mucosa associated lymphoid tissue) and types of metaplasia, as determined from gastric antrum and/or corpus sections (84 NG/138 ZA) obtained from 38 patients (NG) and 74 patients (ZA) by histopathology. The updated Sidney grading system (SGS)^53^ was used for classification. Incomp. = incomplete; comp. = complete; int. = intestinal. (**b**) Percentages of patients with the diagnosis erosion, ulcer or cancer within the patient cohort. The diagnosis was given by the gastroenterologists in Nigeria and South Africa, but could not be confirmed by histology due to a limited number of biopsies. (**c**) The figure shows box and whisker plots (5–95% percentiles) for the presence of active or chronic inflammation. Inflammation was scored according to the SGS with a range from 0 to 3. The mean value is marked by a cross.

As described above, the gastroenterological examination found moderate numbers of patients presenting with symptoms of chronic gastritis. Generally, the rate of gastric complications was slightly higher in Nigeria than in South Africa, as determined by histopathological examinations (Fig. 3a). By endoscopy erosion were the most frequent diagnosis for patients in Nigeria (NG: 43.6%; ZA: 4%) and notably significantly higher in Nigerian than in South African patients (Fig. 3b). Furthermore, gastroenterological diagnosis identified gastric ulcers in both countries (NG: 15.4%; ZA: 9.3%), whereas cancer cases were reported in Nigeria only (2.6%) (Fig. 3b). However, they have not been verified by histopathology due to restrictions in the number of biopsies. There was no statistical association between the diagnosis of erosion or ulcer in South African patients with the phylogenetic profile of the patient’s isolates (e.g. hpAfrica1 or hpAfrica2), or with the ability of the respective isolate to inject CagA into the host cell. When we compared the *H. pylori* positive study population to the *H. pylori* negative participants we found that among the latter patients erosions were also most frequent (NG: 35.4%; ZA: 5.1%), followed by ulcers (NG: 16.5%; ZA: 12.1%). Cancer cases were rare (NG: 0.8%) or absent (ZA). There was no association between the intake of antibiotics, proton pump inhibitors and NSAID with the presence of ulcers, erosions or cancer. This association was tested for all patients regardless of their *H. pylori* infection status and for the combination of positive *H. pylori* infection plus medication intake (data not shown).

For further analysis we subjected stomach biopsies from the patients with positive *H. pylori* isolation to pathohistological examination. Due to autolysis or missing biopsies in Nigeria 84 biopsies from 38 patients and 138 biopsies from 74 patients in South Africa were analyzed. From each included patient at least one biopsy from the antrum and one from the corpus part of the stomach were available. As also reported earlier^22^, the disease outcome was very mild, but unexpectedly, there was no significant difference between patients from Nigeria and South Africa, which was surprising. In our cohorts of both countries, no patients with atrophy, dysplasia, cancer, erosion or ulcers were detected histologically. There was also no significant difference in activity or chronicity of disease between Nigerian and South African patients (Fig. 3c).

### Patients from Nigeria and South Africa harbor *H. pylori* with different phylogenetic profiles

**Figure 4:**
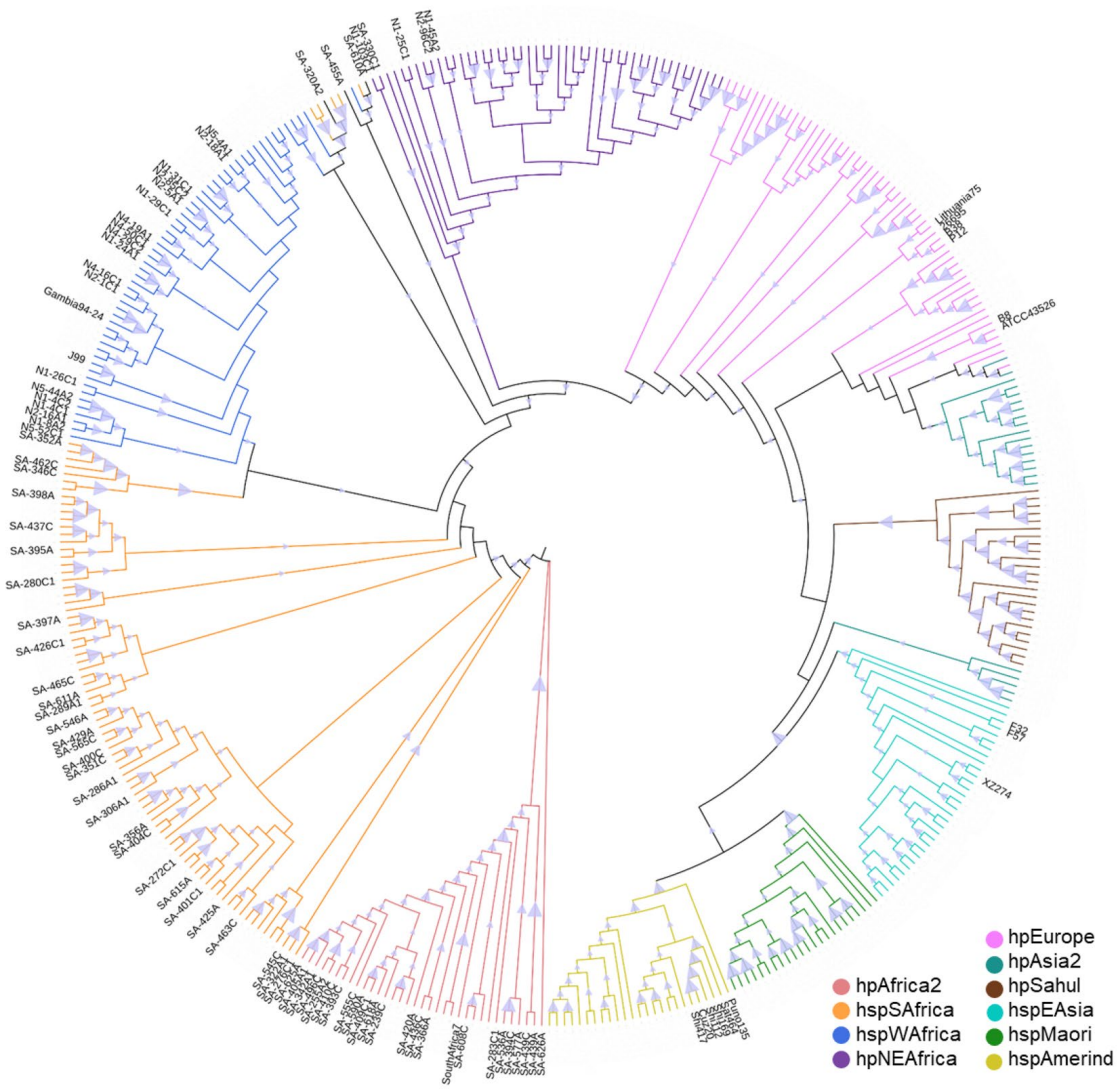
Phylogenetic tree of the isolated *H. pylori* strains. The evolutionary history was computed using the Neighbor-Joining method with 500 bootstrap replicates. The evolutionary distances were computed using the Kimura-2 method. This analysis involved 408 nucleotide sequences. Evolutionary analyses were conducted in MEGA X^48^ (https://www.megasoftware.net/). References were taken from the *Helicobacter pylori* MLST Databases. Reference strains are not annotated but colour-coded. The triangle displays bootstrap values from 0 to 1 drawn to scale.

Knowing from previous studies that *H. pylori* isolates from the southern regions of Nigeria are mainly of hspWestAfrica subtype^22^, and therefore hpAfrica1, we analyzed 20 representative isolates. Except for four patients, whose isolates were of hpNEAfrica origin, all isolates clustered into the expected hspWestAfrica group, as determined by multilocus sequence typing (MLST) (Fig. 4). One of the patients with an isolate originating from Northeast Africa had a history of living in Niger and Mali. Thus, the phylogenetic profiles of Nigerian strains behaved as expected and described before. We next analyzed 56 isolates from South African patients comprising an ethnically more heterogeneous study population as compared to the Nigerian patients and little was known about the phylogeny of *H. pylori* in South Africa. Of these 56 isolates tested, 24 were PCR-negative for the presence of the *cagA*-gene, suggesting they are Type-2 strains^21^ whereas 32 isolates carried a *cagA* gene. As expected, most of the isolates lacking the *cagA* gene fell into the group of hpAfrica2 strains (68.8%), while the rest clustered among hpAfrica1 strains (Fig. 4). Of the 56 isolates, 22 (39.3%) clustered into the hpAfrica2 branch, 30 (53.6%) represent hspSouthAfrica and 1 (1.8%) hpNEAfrica. Three isolates (5.3°%) (SA-610A, SA-455A, SA-322A_2_) clustered into a branch that is clearly hpAfrica1, but includes traits from hspWestAfrica and hspSouthAfrica (Fig. 4) and therefore we were not able to classify these isolates into a distinct subpopulation.

### Virulence factors vary significantly between *H. pylori* isolates from Nigeria and South Africa

As mentioned in “demographic data” more than one isolate per patient was analyzed, but not all isolates could be successfully transferred to the analyzing lab in Munich, limiting the study of the virulence factors to isolates from 39/75 patients, respectively. To avoid statistical bias by including the same isolates twice, we evaluated all isolates with RAPD analysis. Except for three patients, all patients harbored RAPD-identical strains. When the isolates were identical, only one isolate per patient was included. Three patients harbored non-identical strains, therefore all RAPD non-identical strains were included, resulting in 41/77 isolates to be analyzed.

**Figure 5:**
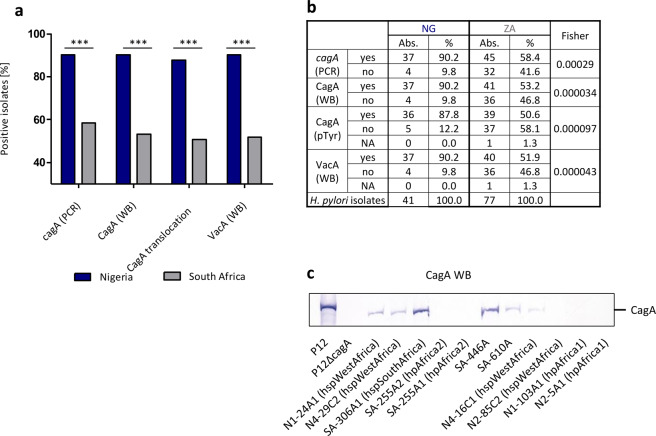
The expression of virulence factors CagA and VacA differs between Nigerian and South African isolates. (**a**) Percentages of patient isolates positive for the presence of *cagA* (as determined by PCR), the expression of CagA and VacA (as determined by western blotting (WB)) or functional CagA translocation as determined by tyrosine phosphorylation assay (pTyr). Groups were compared using Fisher’s exact tests. *p < 0.05, **p < 0.01, ***p < 0.001. (**b**) Statistical analysis of CagA and VacA results. (**c**) Western blot showing the presen**c**e or absence of CagA protein in a set of Nigerian or South African *H. pylori* isolates compared to *H. pylori* P12 reference strain.

**Figure 6:**
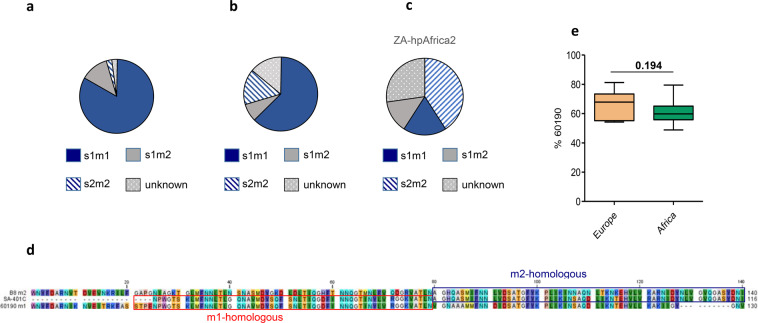
VacA mosaicism differs significantly between Nigerian and South African *H. pylori* isolates. (**a**) *vacA* mosaicism of isolates obtained from Nigerian patients. (**b**) *vacA* mosaicism of isolates from South Africa. (**c**) Representation of *vacA* mosaicism of a sub-cohort of hpAfrica2 isolates from South Africa. (**d**) Depicted is the amino acid sequence in the mid-region of the isolate SA-401C and the m1-reference sequence of 60190 (red box) and the m2-reference sequence of B8 (blue box) demonstrating that SA-401C is a chimera. (**e**) Depicted is the extent of induced cell vacuoles in HeLa cells relative to *H. pylori* control strain 60190. Compared are 11 strains isolated from Europe with 6 isolates from Nigeria and 4 isolates from South Africa in three independent experiments. All isolates from Africa were of *vacA* subtype s1m1. P12, 60190 and P12∆*vacA* served as controls.

We evaluated the isolated strains by PCR and Western blotting for the presence of *cagA* and *vacA* (Fig. 5a,b) and also performed functional assays for the translocation and phosphorylation of CagA into host cells. The frequent presence of hpAfrica2 subtypes in South Africa resulted in a significant difference in expression of *vacA* and *cagA*. While all isolates of hpAfrica2 origin were negative for *cagA*, the *vacA* subtypes showed a mixed phenotype (Figs. 5a,b, 6c).

Interestingly, four of 77 isolates from South Africa were positive for *cagA* PCR, but did not express CagA as detected by Western Blot (SA-289A_1_, SA-330C, SA-378A; SA-480C). Sequencing *cagA* we found frameshift mutations at different sites in SA-289A_1_, SA-330C and a SNP leading to a stop codon in SA-480C. Three hpAfrica1 isolates lacked the *cag*PAI, as determined by Western blotting and sequencing (N2-85C_2_; N1-103A_1_C_1_; N2-5A_1_) (Fig. 5c), whereas eight isolates from South Africa, which were all hpAfrica2, also lacked the complete *cag*PAI. Two isolates from South Africa and one from Nigeria were *cag*A PCR and WB positive, but not able to functionally inject CagA into the host cell as determined via AGS tyrosine phosphorylation assay (SA-446A, SA-610A, N4-16C_1_). Sequencing of *cagA* in these isolates was inconspicuous, suggesting a regulatory mechanism, or mutations in other parts of the *cag*PAI (data not shown).

Another virulence-associated factor implicated in the formation of duodenal ulcers is the *dupA* locus*. H. pylori* solates from WestAfrican origin carry this locus on an integrating conjugative element of the type ICE*Hptfs4a/b*^29^. It was previously shown that this element lacks the 5′-region of *dupA* in isolates of West African origin, because of a truncation, which is shortening the island by around 3.8 kb at the right junction^29^. To compare between the cohorts, we utilized PCR analysis and amplified a DNA fragment spanning this truncation, or the 5′ region of non-truncated *dupA*. The 3.8 kb truncation was present in 82.4% of isolates from Nigeria, but only in 50.7% of isolates from South Africa. Comparing this result with the phylogenetic heritage of the isolates revealed that all isolates with a truncated ICE*Hptfs4* were of hpAfrica1 subtype. Of the analyzed 22 hpAfrica2 isolates, 16 had a full-length *dupA* gene, and in 6 isolates we could not amplify this region. Furthermore, we detected 6 isolates that showed evidence of both *dupA* forms, thus indicating the presence of more than one integrating conjugative element, or at least parts of it.

### Intensive VacA mosaicism in African *H. pylori* isolates and m1-m2 *vacA* chimeras

In order to judge the role of *vacA* and its contribution to virulence, we further evaluated *vacA* mosaicisms in the obtained isolates. Sequence variations in *vacA* gene mosaics differed substantially between the isolates from Nigeria and South Africa (p = 0.01303), most likely attributable to the presence of hpAfrica2 isolates in South Africa (Fig. 6a,b). The majority of Nigerian isolates (82.9%) harbored an s1m1 region, whereas the s1m2 (12.2%) and the s2m2 sequences (2.4%) were less frequent. In South African strains the s1m1 form was dominant as well (62.3%), albeit lower than for Nigerian strains, followed by s2m2 (15.6%) and s1m2, (7.8%) sequences. Notably, a separate analysis of the hpAfrica2 isolates (22) revealed the highest number of s2m2 genotypes (40.9%), while s1/m1 (18.2%) and s1/m2 (13.6%) were less abundant (Fig. 6c). Furthermore, one isolate, SA-401C, represented a chimera of m1 and m2 *vacA* regions, as recently described^30^ (Fig. 6d). An s1b subtype (92.7% versus 88.9%, respectively) dominated in Nigerian as well as in South African strains the s-region subtypes. Remarkably, from 11 South African isolates (14.3%) it was not possible to determine the subtype of either m- or s-region.

We next analyzed the vacuolating activity of both strain cohorts using a quantitative vacuolization assays as previously described^31,32^ and compared cytotoxic activity of African strains with European isolates. Ten randomly chosen isolates from Africa (s1m1 phenotype) were compared to 11 strains of European origin. As a result, the median vacuolating cytotoxicity of the African isolates was slightly lower as compared to the European isolates, but the difference was not significant (Fig. 6e).

### Antibiotic resistance as a common problem in Nigeria and South Africa

**Figure 7:**
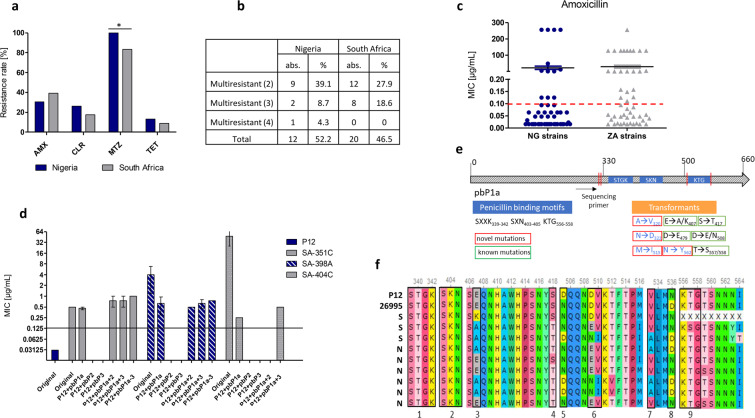
Antibiotic resistance in African isolates. (**a**) Rates of resistance against commonly used antibiotics amoxicillin (AMX), clarithromycin (CLR), metronidazole (MTZ) and tetracycline (TET). 23 patient isolates from Nigeria and 46 patient isolates from South Africa were evaluated. (**b**) Summary of isolates with multiple resistances. (**c**) Distribution of MIC values against amoxicillin of independent *H. pylori* isolates as determined by E-test. (**d**) MIC values of amoxicillin-sensitive P12 strain transformed with various PCR amplification products of *pbp1a*, *pbp2* and *pbp3* and their combinations generated from genomic DNA of amoxicillin-resistant African isolates. Displayed are the mean MIC values of three independent transformation experiments. MIC values were evaluated in two independent two-fold dilution growth curve experiments. The black vertical line marks the cut-off value for resistance (>0.125 mg/L). Empty bar mean that no transformants were obtained in the experiments. (**e**) Schematic representation of *pbp1* with location of penicillin binding motifs and the positions of mutations obtained from amoxicillin-resistant transformants. Novel so far not described mutations are boxed in red, already known and published mutations are boxed in green. (**f**) Alterations in sequences of *pbp1a* in amoxicillin-resistant isolates from Nigeria (N) and South Africa (S) as compared to P12 and 26695 reference strains. Positions 1, 2 and 9 indicated below the sequences mark the penicillin binding motifs.

Antibiotic resistance is a known problem in developing countries with high disease burden and wide access to antibiotics (over-the-counter-policy). We evaluated resistance rates of Nigerian and South African *H. pylori* isolates for commonly used antibiotic drugs amoxicillin, clarithromycin, metronidazole and tetracycline by E-test. Resistance rates varied between substance classes with the highest rate for metronidazole (NG: 100%; ZA: 83.3%) and the lowest for tetracycline (NG: 13%; ZA: 8.7%) respectively (Fig. 7a). For Nigeria 52.5% of isolates were resistant against more than one antibiotic substance with 4.3% being resistant against all four tested antibiotics (Fig. 7b). In South Africa, we found 46.5% of isolates being resistant against more than one antimicrobial substance, but all isolates were susceptible to at least one of the tested drugs. Patients with previous treatment with antibiotics (5/23 patients in Nigeria; 1/46 patients in South Africa) did not show higher resistance rates against one or multiple antibiotics.

### Low and high level of amoxicillin resistance, alterations in penicillin binding proteins, but no β-lactamase activity

After determining surprisingly high resistance rates against amoxicillin with high MIC values, we tried to evaluate possible mechanisms. An overview showing the MIC levels of individual *H. pylori* isolates against amoxicillin from the tested strains from Nigeria and South Africa showed a similar distribution of low-resistance and high-resistance strains in both countries (Fig. 7c). We amplified the genes for penicillin binding proteins 1a, 2 and 3 and transformed corresponding PCR products singularly and in all possible combinations into the sensitive *H. pylori* strain P12. To verify MIC values we determined growth curves in the presence of two-fold dilutions of amoxicillin. In general, the obtained transformants displayed only a low-level resistance against amoxicillin and did not reach resistance levels of the original patient isolates (Fig. 7d). For a successful generation of resistance transfer of *pbp1a* from the resistant strain was necessary, whereas other *pbp* genes, or other unrelated control DNA fragments did not result in an amoxicillin resistant phenotype. We sequenced *pbp1a* of representative transformed P12 isolates and found multiple alterations, but no unifying feature for all resistant isolates (Fig. 7e,f), suggesting that low-level amoxicillin resistance is generated by a cumulative effect of multiple sequence alterations in *pbp1*, as reported previously^33–36^. As postulated by other research groups *H. pylori* high-level amoxicillin resistance may be due to a β-lactamase encoded in *blaTEM-1*^37^. To test for β-lactamase activity we investigated resistant isolates using a commercially available Nitrocefin test. None of the isolates revealed a detectable β-lactamase activity. To control for other mutations or genetic alterations not encoded in penicillin binding proteins we also transformed whole genomic DNA of highly amoxicillin-resistant clinical isolates (MIC > 256 µg/ml) into the sensitive P12 strain. However, transformants with low-level resistance were readily obtained, but no transformants displaying MIC values >2 µg/ml could be observed. In conclusion, this suggests that the mechanism for amoxicillin resistance in *H. pylori* is probably not encoded by a single gene, but might be the result of multiple allelic alterations.

## Discussion

The prevalence of *H. pylori* has been declining in many highly industrialized countries of the Western world, whereas it stayed constant at a relatively high level in certain developing countries. This has major implications on the sequelae associated with an *H. pylori* infection, including peptic ulcer disease and gastric cancer. In this study, we intended to get more insights in *H. pylori* patient isolates from Nigeria versus South Africa and to compare their phylogenetic profiles, their virulence factors, and their antibiotic resistance profiles, known to be a common problem in Nigeria and South Africa. To avoid statistical bias or overestimation of certain characteristics, we decided to interpret only one *H. pylori* isolate per patient when the isolated strains were the same as determined by RAPD PCR analysis. This procedure resulted in a relatively low number of examined isolates. We adjusted statistic testing to the Fisher’s exact test that is suitable for small numbers of cases. All patients of the Nigeria and the South Africa cohorts are listed in Fig. 2b and differentiated according to gender and infection status (column absolute), and the cases for which *H. pylori* isolates could be obtained (column: patients with positive isolation). Only these latter patients and patient isolates, for which all tests have been performed, have been included into the evaluation of our results. We also assume that the set of isolates tested in detail is representative for the whole 1121 patients included in the study, since the gender and age distribution of the patient set with isolates studied is in a similar range as that of all patients. The relatively low number of isolates that could be analyzed in the lab as compared to the relatively high number of participants has multiple reasons. Due to the fastidious nature of *H. pylori*, isolation rates have been reported to vary greatly (30–70%) between laboratories^38,39^. In Nigeria it was not possible to isolate *H. pylori* directly at the sampling site and with difficult infrastructure conditions there were significant delays in the isolation procedure. Isolates were then shipped to Munich causing a significant proportion of isolates that could not be regrown from the transport medium due to a long transport time.

By comparing the disease outcome of Nigerian and South African patients and the patient isolates, it was obvious that the number of severe complications of *H. pylori* infected patients, like erosions (NG: 43.6%; ZA: 4.0%), ulcers (NG:15.4%; ZA: 9.3%) or cancer (NG: 2.6%; ZA: 0%) was lower in South African patients than in Nigerians. A low gastric cancer rate in the African countries might be explained by a generally lower life expectancy as compared to many other countries. Furthermore, it could be speculated that one reason for that might be the high number of hpAfrica2 strains in South Africa, which lack the main virulence factor, the *cag*PAI and are supposed to mainly express the less virulent *vacA* subtype s2m2. However, our data show now that more pathogenic s1m1 and s1m2 *vacA* subtypes (together 31%) are found in hpAfrica2 strains, which is unexpected and challenges the hypothesis that preferentially *H. pylori* type I strains, carrying a functional *cag*PAI, express the s1m1 *vacA*^19^. Our data show that *vacA* s1m1/m2 is well compatible with the absence of the *cagPAI* and suggests that in such ancient, *cag*PAI deficient strains both types, s1m1/m2 and s2m2 *vacA* genes were existent. With acquisition of the *cagPAI* during evolution by *H. pylori* a selection pressure might have favored the co-expression of s1m1 *vacA* subtypes with these strains.

We were not able to link the association statistically, because the case numbers with gastroenterological disease were in general quite low. The pathology examination could not confirm erosions or ulcerations and it remained unclear if this was due to sampling errors during gastroscopy (e.g. sampling in the wrong areas), or interobserver variations. However, in this context similar complications have already been described in the past^22,40^.

The continuous increase of antibiotic resistance levels in bacteria is a worldwide problem^41,42^, but cannot be overemphasized in developing countries with difficult access to professional health care and open sale of antibiotics. In our study, antibiotic intake within the past three months did not directly influence resistance, but 52.12% (NG) and 46.51% (ZA) of isolates were resistant against more than one antibiotic. Overall, the resistance rates we observed in our study were well in the range of a recent meta-analysis published by Jaka *et al*.^43^.

Notably, only 15% (NG) and 1.3% (ZA) of all patients included in this study declared to be pretreated with antibiotics in the past. All patients with positive UBT or PCR result were considered as *H. pylori* infected, but we included only patients with successful *H. pylori* isolation into the further study. To control for a bias caused by positive selection of untreated patients, we next compared all patients with positive UBT or PCR result for *H. pylori*. From this cohort 16.5% (NG) and 3.9% (ZA) of patients, respectively, had declared to be pretreated with antibiotics (data not shown), which is comparable to the overall patient cohort and argued against a bias in our studied patient cohort.

Since antibiotic resistance against amoxicillin has been discussed quite controversial and the reported resistance rates vary quite drastically^43^, we decided to study the responsible mechanisms. None of the isolates tested exhibited a β-lactamase activity, as originally reported by Tseng *et al*. for amoxicillin resistant *H. pylori* strains^37^. We next generated *pbp1a*, *pbp2* and *pbp3* DNA fragments from highly amoxicillin-resistant *H. pylori* isolates (Fig. 7e). Transformation of *pbp1a*, but not *pbp2* or *pbp3* into the sensitive P12 strain resulted in amoxicillin-resistant transformants, although the full resistance level of the original isolates was not obtained, which is different to previously reported results^44^. We found multiple alterations in the sequence of *pbp1a*, but none of the alterations was a unique feature to all resistant isolates (Fig. 7e). Interestingly the penicillin binding motifs SXXK_339–342_, SXN_403–405_ and KTG_556–558_ were commonly not affected (except for one isolate with a threonine to serine substitution in KTG_556–558_), but the region AA 500–550 in close proximity to the KTG motif was the sequence with the highest variation (Fig. 7e,f). Taken together our results suggest that single mutations in the *pbp1a* gene result in a low-level amoxicillin resistance of *H. pylori*, but other mechanism(s) conferring a high-level amoxicillin resistance are so far unknown.

## Methods

### Study design

Patients were recruited between November 2015 and July 2018 in different hospitals in Nigeria (NG) and South Africa (ZA). In Nigeria five participating hospitals located in Lagos, one each in Ibadan, in Ile-Ife and in Jos. Patients in South Africa were recruited from two different hospitals in Johannesburg and Port Elisabeth. Each patient filled a questionnaire and signed a consent form and a gastroenterological diagnosis was provided by the gastroenterologists in charge.

Urea breath test (UBT), which, besides a PCR or a nested PCR approach, is considered as the gold standard in *H. pylori* diagnostics for African countries, was performed^22^, and six biopsies (three from the antrum and three from the corpus) were taken from each patient. One antrum and one corpus biopsy were used for isolation of *H. pylori*, while the other two biopsies were used for extraction of DNA and histological analysis.

The inclusion criteria comprised the treatment in one of the mentioned hospitals and the patient agreement to participate in this study. The recruitment, the gastroenterological examination, the UBT, the fixation of the biopsies and the isolation of the strains were performed in the hospitals in Nigeria and South Africa and the corresponding laboratories of the Nigerian Institute of Medical Research, Lagos and University of Fort Hare, Alice. Throughout the course of this study, patient isolates, questionnaires and forms with gastroenterological results were transferred to the Max von Pettenkofer-Institut of the LMU in Munich, Germany. A more intensive biochemical, immunological and molecular characterization of isolates as well as a histological grading of biopsy material was performed in Munich. These assays included the determination of the status of antibiotic resistance levels (MIC tests) the presence or absence of genes encoding for *H. pylori* virulence factors by polymerase chain reaction (*cagA, vacA*), as well as the detection of expression and function of major virulence factors like CagA and VacA by western blotting, CagA translocation and vacuolization assays. A statistical analysis was performed to determine correlations between *H. pylori* virulence, risk factors and disease outcome.

### Urea breath test

The urea breath test (UBT) was obtained from Tri-Med Distributors in Perth, Australia. UBT was performed as described previously^22^. Briefly, patients fasted either overnight or for a minimum of four hours. A capsule containing a known amount of ^14^C-labelled urea was provided to patients to drink with 30 mL of water, followed after 3 minutes by a further 30 ml of water. After further 7 minutes a breath sample is collected in a sterilized mylar balloon. Following contact between ^14^C-labelled urea and stomach-resident *H. pylori*, the molecule is hydrolyzed into ^14^C-carbon dioxide and ammonia. The carbon dioxide is then exhaled by the patient. The contents of the balloon were dissolved into breath collection fluid and then liquid scintillation fluid was added to quantify the degree of ^14^C in the sample. Scintillation values below 50 DPM (disintegrations per minute) were considered to be negative, values over 200 DPM as positive and all values between as “borderline”.

### *H. pylori* isolation

The isolation of *H. pylori* from biopsy specimens was performed as described previously^22^. Briefly, biopsy specimens were aseptically rolled over the surface of Columbia blood agar base plates under a biological safety cabinet (Thermo Fischer Scientific). The agar (Oxoid CM0331) was supplemented with 7% horse serum (Oxoid SR0048), 1% vitamin mix (Isovitale-X), and an *H. pylori* selective supplement (Dent, SR0147E Oxoid) comprising of amphotericin B (2.5 mg), trimethoprim (2.5 mg), vancomycin (5.0 mg), and cefsulodin (2.5 mg). The plates were incubated at 37 °C in an atmosphere of 85% N_2_, 10% CO_2_ and 5% O_2_ for 4–10 days. *H. pylori* colonies were identified as small, round, translucent, Gram-negative bacteria and positive for catalase, oxidase and urease tests. The confirmed isolates were frozen in Brucella broth containing 20% glycerol and stored at −80 °C for future use.

### Transport of *H. pylori* strains

For transport of the isolates from Nigeria or South Africa to Munich (Germany), strains were cultured on GC agar serum plates, passaged at least twice and transferred into Portagerm Pylori (bioMérieux SA Mercy L’Etoile France) medium for shipment at room temperature, as described previously^22^.

### PCR and MLST

PCR was performed in 25 µl reaction mixtures, consisting of 1X PCR buffer, magnesium chloride (1.5 mM), dNTP (200 μM), primer (10 pmol) and 1U Taq DNA polymerase (Pan-Biotech GmbH). Amplification was carried out in a Peqlab Thermocycler using the following cycling parameters: initial denaturation at 95 °C for 5 min, followed by 35 cycles of 95 °C for 30 sec, 52 °C for 30 sec and 72 °C according to amplicon length (1 min per 1000 bp). This was followed by a final extension of 72 °C for 10 min. The *hpWAfrica* strain J99^45^ and the *hpEurope* strain P12^46^ were used as control strains. Primers are listed in Table 1. For strain phylogeny, multilocus sequence typing analysis was used^47^. Partial sequences of 7 housekeeping genes were obtained by Sanger sequencing after purifying PCR products with Illustria GFX PCR DNA and gel band purification kit. The resulting sequences were assembled and aligned with the corresponding sequences from *H. pylori* reference strains from the MLST database, using the Muscle algorithm within MEGA X^48^. Phylogenetic trees were constructed and tested by neighbor-joining method using the Kimura 2-parameter model of nucleotide substitution, and 500 bootstrap replications.

**Table 1:** Primer sequences.

Target gene	Sense primer	Antisense primer	Amplicon
cagA	ACCGCTCGAGAACCCTAGTCGGTAATGGG	ATATCGATTTAAGCCAATTTTTGATTCCTTG	500 bp
cagA (full length)	CCATCGATGG TAAAAATGTG AATCGT (454)	CAGGTACCGC GGCCGCTTAA GATTTTTGGA AACCAC (1314)	3658 bp
vacA s1	CTGCTTGAATGCGCCAAAC	ATGGAAATACAACAAACACAC	259 bp
vacA s2	CTGCTTGAATGCGCCAAAC	ATGGAAATACAACAAACACAC	286 bp
vacA m1	GGTCAAAATGCGGTCATGG	CCATTGGTACCTGTAGAAAC	290 bp
vacA m2	CATAACTAGCGCCTTGCAC	CATAACTAGCGCCTTGCAC	352 bp
atpA	GGACTAGCGTTAAACGCACG	CTTGAAACCGACAAGCCCAC	841 bp
efp	GGCAATTTGGATGAGCGAGCTC	CTTCACCTTTTCAAGATACTC	559 bp
mutY	GTGGTTGTAGYTGGAAACTTTACAC	CTTAAGCGTGTGTYTTTCTAGG	676 bp
ppa	GGAGATTGCAATGAATTTAGA	GTGGGGTTAARATCGTTAAATTG	706 bp
trpC	TAGAATGCAAAAAAGCATCGCCCTC	TAAGCCCGCACACTTTATTTTCGCC	633 bp
ureI	AGGTTATTCGTAAGGTGCG	GTTTAAATCCCTTAGATTGCC	686 bp
yphC	CACGCCTATTTTTTTGACTAAAAAC	CATTYACCCTCCCAATGATGC	734 bp
pbp1a	GATTGTCATAGGGTTGTTAGC (PP-14)	GGGTTCTTCGCTATCGTC (PP-15)	1928 bp
pbp2	GTCTTCGCTATAAGCTTTTG (PP-54)	CTCATAGAGTTTGTTGCTC (PP-55)	1745 bp
pbp3	TGATCCTTACTTCAACCCA (PP-56)	GTTTTGAATCGCAATAGAGGG (PP-57)	1797 bp
dupA	CTACAATATAGCTCTCAAAAG(WS539)	AGCAATAAAACGCTTAAAAGTCTC(WS606)	2935 bp
dupA	GTATTCCTAGCCAATATTCTTTAG(WS677)	AAAAATTTAGGCTCAAAGTCTG (WS678)	597 bp

### Western blotting

Sodium dodecyl sulfate-polyacrylamide gel electrophoresis (SDS-PAGE) and Western blotting was performed as described^47^ using polyvinylidene difluoride (PVDF) filters blocked with 3% BSA in TBS (50 mM Tris-HCl, pH 7.5, 150 mM NaCl), 0.1% (v/v) Tween 20. Rabbit polyclonal antisera against CagA and VacA have been described previously^49^. Alkaline phosphatase-conjugated protein A or horseradish peroxidase-conjugated anti-rabbit IgG antiserum was used to visualize bound antibody.

### Phosphorylation of translocated CagA

To determine capability of CagA-translocation AGS cells were infected with *H. pylori* strains and phosphotyrosine immunoblotting were performed^10^. Briefly, cells were infected with bacteria adjusted to OD_550_ 0.2 for 4 h at 37 °C, washed three times and suspended in PBS containing 1 mM EDTA, 1 mM Na_3_VO_4_, 1 mM PMSF, 10 µg/ml leupeptin, and 10 µg/ml pepstatin. Cells were then collected by centrifugation and resuspended in sample buffer. Tyrosine-phosphorylated proteins were analyzed by immunoblotting with the phosphotyrosine antiserum PY99 (Santa Cruz Biotechnology).

### Vacuolization assay

To determine cytotoxicity, a vacuolization assay, as described previously was performed^32^. Briefly HeLa cells were grown to 70% confluency, infected with OD_550_ 0.1 and incubated for four hours at 37 °C and 10% CO_2_. After incubation with NH_4_Cl for another hour, cells were stained with neutral red and washed twice with PBS + 15% FCS. Cells were then incubated with 70% ethanol+ 0.37% HCl for 5 minutes and the extinction measured at 534 nm in a TECAN plate reader. Values were normalized to the control strain 60190. A *H. pylori* P12∆*vacA* mutant^31^ served as negative control.

### MIC test

The minimal inhibitory concentration was obtained using E-test strips and performed according to manufacturer’s instructions (Liofilchem, Italy) and as described earlier^22^. *H. pylori* isolates were grown as a liquid culture in brucella broth + 10% fetal calf serum (FCS) for 3–4 hours until OD_550_ 1.0. 400 µl of the liquid culture was transferred to a GC agar serum plate and allowed to dry for several hours. An antibiotic strip was placed on the plate. Antimicrobial agent concentrations ranged from 0.016 to 256 µg/mL. After three days incubation the zone of inhibition was measured under consideration of recommended MIC breakpoints (EUCAST Clinical Breakpoint Table v. 8.0, valid from 2018-01-01): amoxicillin >0.125 µg/mL, clarithromycin >0.5 µg/mL, tetracycline >1 µg/mL, metronidazole >8 µg/mL. Every isolate was tested twice independently with E-test.

### Transformation

PCR products of penicillin binding proteins *pbp1a*, *pbp2* and *pbp3* of resistant isolates were generated (see primer listed in Table 1) and transformed into the sensitive *H. pylori* strain P12 by natural transformation, as described previously^50^. *H. pylori* transformants were selected on serum agar plates containing 1 mg/L amoxicillin (Sigma-Aldrich). Clones were frozen in brucella broth containing 20% glycerol and 10% FCS, stored at −80 °C and plated again on GC agar plates containing 1 mg/L amoxicillin.

### Growth curves

MIC values of clones were evaluated using growth curve assays, as described earlier^51^. *H. pylori* were diluted to an OD_550_ 0.075 and subcultured in 96-well microtiter plates (clear, flat-bottom, Costar, Corning Inc.), which were then sealed with a gas-permeable membrane (Breathe-Easy sealing membrane, Diversified Biotech). Plates were incubated at 37 °C under microaerobic conditions (while shaking) in the Clariostar plate reader (BMG Labtech). Optical density was automatically monitored and measured every five minutes. Growth curves were analyzed and processed with MARS Data Analysis software 3.10 R5 (BMG Labtech). Percental growth was expressed as [(ΔODsample/ΔODcontrol) x 100], while ΔOD corresponded to “OD_stationary phase_ − OD_lag phase_”.

### Nitrocefin tests

A Nitrocefin test was used to evaluate for β-lactamase activity. Nitrocefin is a chromogenic cephalosporin, with a β-lactam ring. Upon oxidation by β-lactamase color is rendered from yellow to red. Nitrocefin disks for rapid detection of β-lactamase enzymes were purchased from Sigma and used as suggested by the manufacturer. Briefly, disks were allowed to equilibrate to room temperature and were humidified with 10 µl of H_2_O_bidest._ Single colonies were streaked directly from GC agar plates on the discs and incubated for 20 minutes to observe a color change from yellow to red. *H. pylori* P12[TEM-1-CagA]^51^ served as positive control and *E. coli* DH5α as negative control.

### Histology

Biopsies were fixed in 4% buffered formalin at the study sites and sent to Munich. Hematoxylin and eosin stained longitudinal paraffin sections of antrum and corpus (one section per location) were graded according to the updated Sydney System^52^ by pathologists in Munich on the intensity of inflammation, metaplasia, and presence of gastric mucosa associated lymphoid tissue (MALT), as well as the presence and occurrence of atrophies, metaplasia, dysplasia, cancer, erosion and ulcers.

### Ethics

The study was reviewed and approved by the Ethics Committee of the Nigerian Institute of Medical Research (registration number IORG0002656), as well as the Ethical Committee of the Chris Hani Baragwanath Academic Hospital (CHBAH) Soweto Johannesburg (registration number M160228), the University of Fort Hare Alice Eastern Cape South Africa (registration number Rec-270710-028-RA level 01) and the Ludwig-Maximilians-University Munich (registration number 335-08). The study was conducted in line with the Declaration of Helsinki and informed consent was obtained from all study participants.

### Statistical methods

Interrelations between clinical, pathologic, and laboratory findings were investigated bivariately using chi-squared or fishers exact tests (frequencies less than 5 for a statistical event)^22^. Due to the exploratory character of these analyses, all tests were performed on an alpha level of 5% without any correction for multiple testing. P-values depicted in this manuscript value: *p < 0.05, **p < 0.01, ***p < 0.001. All analyses were done with R: A Language and Environment for Statistical (Vienna, Austria).

## Data Availability

The datasets generated during and/or analyzed during the current study are available from the corresponding author on reasonable request.
